# Exploratory MRI and Clinical Marker Analysis of Pustulotic Arthro‐Osteitis Disease Activity From a Phase 3 Brodalumab Trial

**DOI:** 10.1111/1346-8138.70425

**Published:** 2026-08-02

**Authors:** Taiki Nozaki, Manabu Arai, Junko Ochi, Mona Uchida‐Yamada, Masahiro Hashimoto, Mitsuhiro Akiyama, Yuko Kaneko, Yasumasa Kanai, Masahiro Jinzaki

**Affiliations:** ^1^ Department of Radiology Keio University School of Medicine Tokyo Japan; ^2^ Department of Radiology University of Iowa Iowa City Iowa USA; ^3^ Medical Affairs, Kyowa Kirin Co., Ltd. Tokyo Japan; ^4^ Division of Rheumatology, Department of Internal Medicine Keio University School of Medicine Tokyo Japan

**Keywords:** magnetic resonance imaging, osteitis, palmoplantar pustulosis, rheumatology, SAPHO syndrome

## Abstract

Pustulotic arthro‐osteitis (PAO) is a clinically important osteoarticular comorbidity affecting approximately 9%–28% of patients with palmoplantar pustulosis (PPP), yet early recognition and standardized activity assessment remain challenging, particularly in routine dermatology practice. This exploratory analysis evaluated patients with PAO to identify potential indicators of disease activity. A magnetic resonance imaging (MRI)‐based scoring system for the anterior chest wall (ACW) in patients with PAO was developed. This post hoc analysis used data from the PAO subgroup of patients with PPP enrolled in a previous phase 3 trial. Correlations were assessed between clinical indicators—Ankylosing Spondylitis Disease Activity Score, Assessment of SpondyloArthritis international Society Health Index score, physicians' Numeric Rating Scale‐PAO (NRS‐PAO) activity score, and patients' NRS‐PAO pain score—and MRI indicators, including active lesions such as bone marrow edema extent and intensity, and structural lesions in the ACW, spine, and sacroiliac joints (Spondyloarthritis Research Consortium of Canada [SPARCC] score); the correlations between MRI indicators and plain radiographic indicators were also evaluated. Of 20 included patients (mean age: 56.6 years), the majority were female. MRI examinations detected bone marrow edema in all patients. None of the clinical indicators showed a clear association with MRI indicators. There was no clear association between radiographic indicators and the bone marrow edema extent or intensity scores of lesions in the ACW. The spine SPARCC score was positively correlated with the osteosclerosis score of the ACW (|*r*| = 0.70; *p* = 0.0218) and the modified Stoke Ankylosing Spondylitis Spinal Score of the spine (|*r*| = 0.93; *p* < 0.001). The MRI‐based ACW scoring system may provide a feasible method to standardize disease activity assessment in PAO in clinical practice. Further external validation is warranted, and additional clinical indicators are needed to predict disease activity.

**Trial Registration:** NCT04061252, UMIN000055398

## 1 Introduction

Pustulotic arthro‐osteitis (PAO) is a joint comorbidity of palmoplantar pustulosis (PPP) [1], also recognized within the spectrum of Synovitis, Acne, Pustulosis, Hyperostosis, and Osteitis (SAPHO) syndrome [2], and is estimated to affect approximately 9%–28% of patients with PPP [3, 4], highlighting the clinical relevance of PAO in dermatology practice. Although PAO is not classified as a spondyloarthritis [5], it exhibits osteoarticular symptoms similar to those of axial spondyloarthritis [6, 7]. In PAO, osteoarticular involvement primarily affects the axial skeleton, including the anterior chest wall (ACW), spine, and sacroiliac joints, as well as peripheral joints [8, 9]. Notably, pain and swelling often occur in the joints of the ACW, particularly the sternoclavicular and costoclavicular joints [6]. The onset of osteoarticular lesions does not necessarily coincide with skin manifestations, and the severity of these symptoms may not be correlated [9]. Despite these insights, large‐scale epidemiological studies on PAO disease activity, clinical features, and assessment measures are lacking, leaving many aspects of the disease unresolved.

Recently, the modified PAO Japanese diagnostic guidance has recommended early diagnosis to prevent irreversible structural changes that may affect patients' quality‐of‐life [10]. Although previous studies have assessed PAO using pain Visual Analog Scale scores, quality‐of‐life indicators (EuroQol 5‐Dimension), Bath Ankylosing Spondylitis Disease Activity Index, Ankylosing Spondylitis Disease Activity Score (ASDAS), Patient Global Assessment, Disease Activity in Psoriatic Arthritis, and imaging indicators (bone marrow edema, bone erosion, and bone proliferation), their associations with PAO disease activity have not been investigated [11, 12, 13, 14, 15]. In addition, inflammatory laboratory biomarkers may be normal or mildly elevated, making imaging central to both diagnosis and disease activity monitoring [16].

Conventional radiography and computed tomography are useful for depicting structural changes. However, they have limited ability to visualize active inflammatory lesions, whereas magnetic resonance imaging (MRI) can detect bone marrow edema and soft tissue involvement, as well as distinguish active from chronic lesions, without radiation exposure [10]. The modified Japanese diagnostic guidance for PAO recommends joint evaluation using MRI in addition to plain radiography for early diagnosis [10]. The guidance emphasizes the importance of MRI for assessing disease activity (active inflammatory changes), particularly in the ACW, which is often the predominant site of involvement in patients with PAO [10]. Ikumi et al. incorporated MRI findings such as bone marrow edema, soft tissue swelling, and joint inflammation, along with other clinical assessments, to evaluate the effectiveness of a biologic agent in patients with PAO [11]. In another study, Yamamoto Fukuda et al. used MRI assessments (inflammation of synchondrosis, bone marrow edema/osteitis, soft tissue swelling/edema, bone erosion, and bone proliferation) of anterior chest and spine lesions to evaluate treatment efficacy in PAO [14]. However, no standardized method currently exists for evaluating disease activity in the ACW in PAO. Furthermore, while quantitative evaluation and MRI‐based scoring systems such as the Spondyloarthritis Research Consortium of Canada (SPARCC) score are well established for assessing disease activity in the spine and sacroiliac joints in ankylosing spondylitis [17], it remains unclear whether these are appropriate measures for PAO. Although PAO shares some clinical and radiological overlap with ankylosing spondylitis, it has been reported to exhibit distinct imaging patterns and disease characteristics, raising uncertainty about the direct transferability of ankylosing spondylitis–derived activity measures to PAO, particularly for ACW‐dominant disease [18]. The differing clinical features and treatment approaches between ankylosing spondylitis and PAO underscore the need for PAO‐specific indicators. Therefore, it is essential to establish an MRI‐based scoring method to evaluate disease activity in PAO.

Hence, we performed an exploratory analysis using data from a subgroup of patients with PPP and concurrent PAO who were enrolled in a phase 3, randomized, placebo‐controlled trial of brodalumab to identify a method for evaluating PAO disease activity. We also developed an MRI‐based scoring system as a disease activity indicator for ACW lesions in patients with PAO.

## 2 Methods

### 2.1 Study Design and Patient Population

This exploratory analysis was based on a subgroup of patients with PPP and concurrent PAO enrolled in a phase 3, randomized, double‐blind, placebo‐controlled trial (NCT04061252). The details of the study have been published previously [19]. Briefly, the trial included patients aged ≥ 18 to ≤ 70 years diagnosed with PPP (defined as a Palmoplantar Pustulosis Area and Severity Index [PPPASI] total score ≥ 12 and PPPASI severity score for pustules/vesicles on the palms or soles ≥ 2) who were inadequate responders to existing therapies. Patients were enrolled in the trial regardless of whether they had a PAO diagnosis.

At enrollment, a rheumatologist or dermatologist trained in rheumatology assessed each patient to determine PAO eligibility based on predefined criteria. Patients were included in the PAO subgroup if they were inadequate responders to oral nonsteroidal anti‐inflammatory drugs or acetaminophen and had a pain score of ≥ 4 on the PAO Numeric Rating Scale (NRS‐PAO). Inclusion also required the presence of at least 1 of the following symptoms: (1) obvious swelling and tenderness in the ACW or peripheral joints after ruling out other conditions (e.g., osteoarthritis); (2) tenderness without obvious swelling in the ACW or peripheral joints, accompanied by imaging evidence of active inflammatory lesions or structural bone changes (e.g., bone erosion, hyperostosis, or syndesmophytes) at the tender site; or (3) back pain lasting longer than 3 months, with imaging evidence of active inflammatory lesions or structural changes in the spine or sacroiliac joints. Patients with other conditions that could confound the diagnosis of PAO, such as fibromyalgia, ankylosing spondylitis, rheumatoid arthritis, diffuse idiopathic skeletal hyperostosis, reactive arthritis, spondylitis associated with inflammatory bowel disease, or psoriatic arthritis, were excluded from the PAO subgroup.

Imaging examinations were optional in the phase 3 trial. Patients for whom all required imaging and clinical (non‐imaging) indicators for the exploratory analysis were not available were excluded from this analysis.

### 2.2 Assessments

Patients with PPP and concurrent PAO underwent a series of clinical and imaging evaluations. In this exploratory analysis, only baseline data were used.

Clinical indicators included ASDAS with C‐reactive protein (ASDAS‐CRP), Assessment of SpondyloArthritis international Society (ASAS) Health Index score, physicians' NRS‐PAO activity score, and patients' NRS‐PAO pain score [20, 21, 22]. In principle, all assessments were performed consistently by the same physicians throughout the study period. The physicians were board‐certified rheumatologists or dermatologists with specialized training in rheumatology.

MRI scans of the chest and sacroiliac joints were mandatory, whereas spinal MRI scans were optional. MRI scans were performed using either 1.5‐T (for 11/19 [57.9%] patients) or 3.0 T (for 8/19 [42.1%] patients) scanner with appropriate surface coils. The MRI imaging conditions were determined in advance to ensure consistency among images acquired at different facilities as below, and all MRI scans were performed according to these conditions. The imaging data were immediately sent to the data center, where radiologists evaluated the images and issued instructions for parameter adjustments or repeat imaging when scans were deemed unsuitable for evaluation, thereby striving to standardize the imaging process as much as possible. Coronal images of the ACW were obtained with a 3‐mm slice thickness; oblique coronal images of the sacroiliac joints were obtained with a 4‐mm slice thickness. Sagittal images of the spinal cord were obtained from the first cervical vertebra (C1) to the tenth thoracic vertebra (T10) with a 4‐mm slice thickness, and from the eighth thoracic vertebra (T8) to the first sacral vertebra (S1) with a 3‐mm slice thickness. Imaging parameters were as follows: T1‐weighted spin echo (repetition time [TR]: 400–500 ms; echo time [TE]: 10–20 ms) and short‐tau inversion recovery (STIR; TR: 3500–5000 ms; inversion time [TI]: 150–180 ms for 1.5‐T scanners and 220–250 ms for 3.0‐T scanners; TE: 40–60 ms).

MRI of the ACW was used to evaluate the extent and intensity of bone marrow edema as indicators of active lesions, and sclerosis and fatty degeneration as indicators of structural lesions. The evaluation criteria for ACW MRI established in this analysis included imaging of the sternoclavicular joint (clavicular/manubrial side), sternocostal joint (costal/manubrial side), and manubriosternal joint (manubrial/gladiolus side). The sternoclavicular and sternocostal joints were assessed separately on the left and right sides for a total of 8 locations, whereas the manubriosternal joint was assessed as a whole for a total of 2 locations (Figure 1). Imaging was performed using T1‐weighted imaging (T1WI) and STIR sequences. Cross‐sections displaying the most prominent signal abnormalities were selected, and the muscle signal on STIR was used as the reference signal.

**FIGURE 1:**
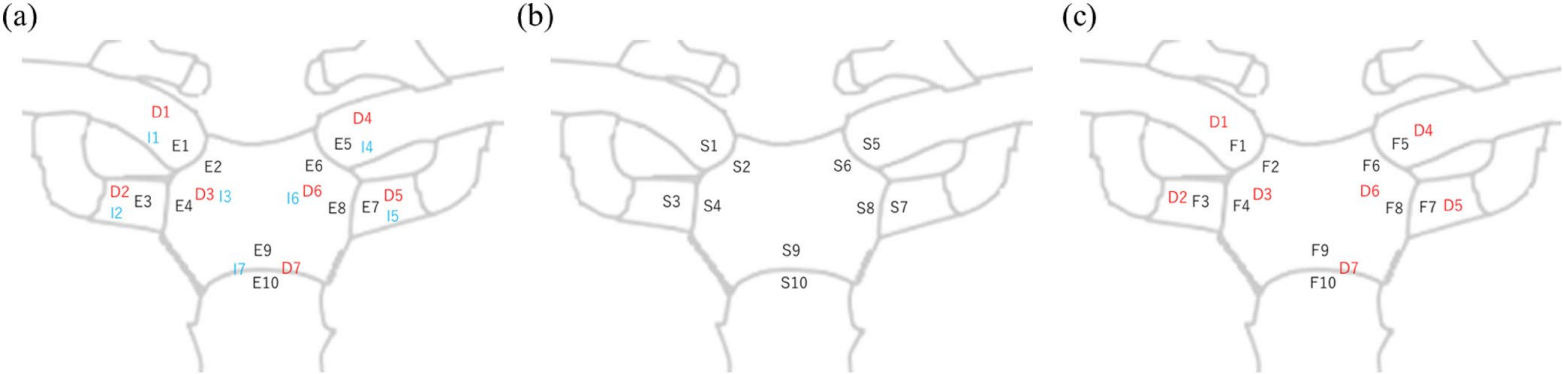
Measurement method for the extent of bone marrow edema (a) as an indicator of active lesions, and sclerosis (b) and fatty degeneration (c) as indicators of structural lesions. D = depth; E = bone marrow edema; F = fatty degeneration; I = intensity; S = sclerosis.

The bone marrow edema extent score (score range: 0–24) was assessed as follows: 1 point was assigned for the presence of edema at each of the 10 designated sites on the STIR image, as shown in Figure 1a. Intensity (high‐signal lesion equal to vessels) and depth (high‐signal area with a long diameter of ≥ 1 cm) were assessed at 7 locations: bilateral clavicle, first rib, manubrium of the sternum, and the midline junction between the manubrium and the body of the sternum (either the manubrium or the sternal body). A total score was calculated based on these assessments (Figure 2).

**FIGURE 2:**
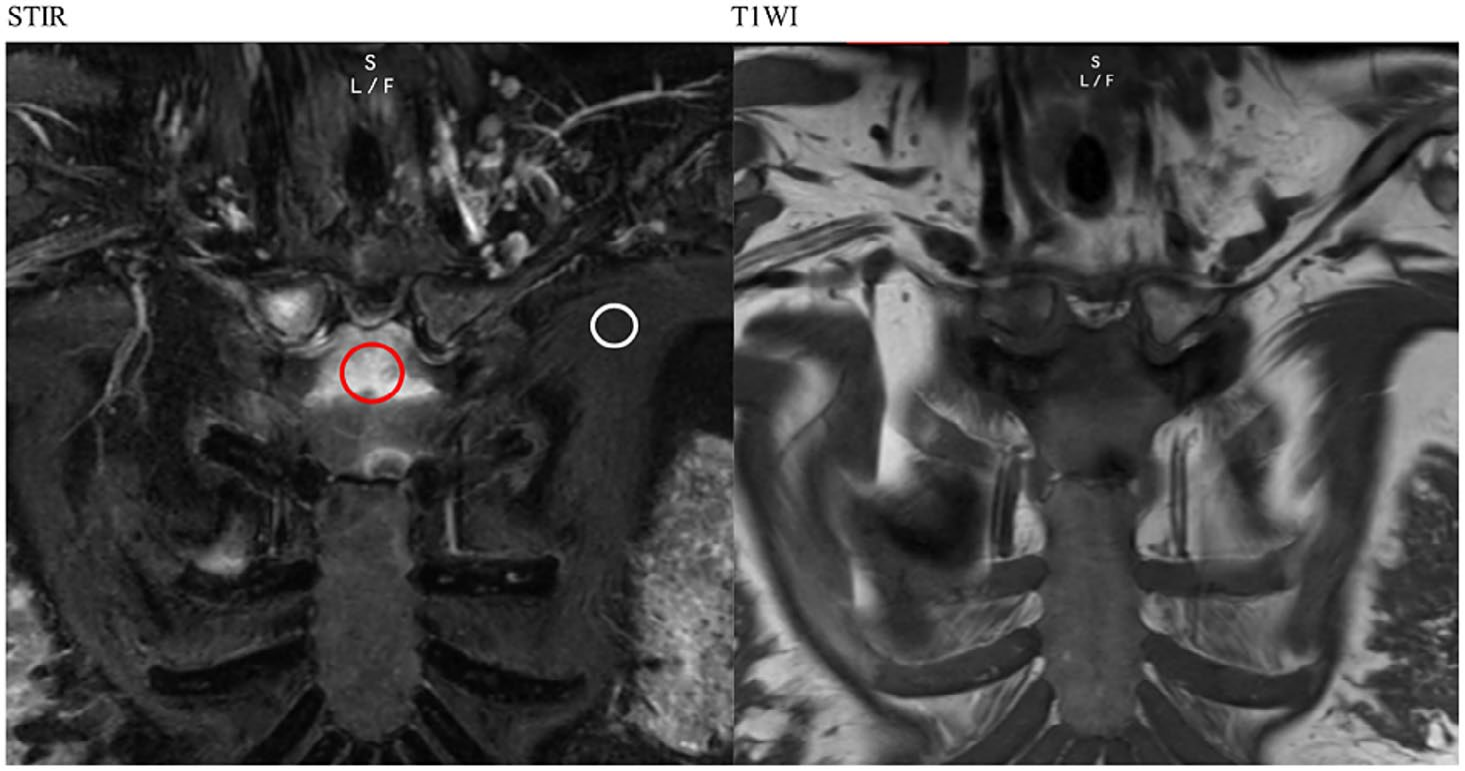
Signal intensity measurements performed on T1WI and STIR images. Signal intensity measurements were performed by identifying anatomical structures on T1WI and placing circular regions of interest on STIR images within areas of bone marrow edema (red circle) and adjacent muscle (white circle). The maximum signal intensity values were used for both tissues. For muscle, easily measurable muscles near the area of interest, such as the pectoralis major or intercostal muscles, were selected. Each measurement was performed 3 times, and the average value was used for analysis. The edema intensity ratio was calculated using the following formula: Edema intensity ratio = Maximum signal intensity of bone marrow edema/maximum signal intensity of muscle. Bone marrow edema extent score = 12 points. Edema: E1, E2, E4, E6, E8, E9. Depth: D1, D3, D6. Intensity: I1, I3, I6. Bone marrow edema intensity ratio = 2468/508 = 4.86. Maximum signal intensity of bone marrow edema = 2468. Maximum signal intensity of muscle = 508. STIR, short‐tau inversion recovery; T1WI, T1‐weighted imaging.

The signal intensity measurements were performed by identifying anatomical structures on T1WI and placing circular regions of interest on STIR images within areas of bone marrow edema and adjacent muscle. The maximum signal intensity values were used for both tissues. For muscle, easily measurable muscles near the area of interest, such as the pectoralis major or intercostal muscles, were selected. Each measurement was performed 3 times, and the average value was used for analysis (Figure 2). The bone marrow edema intensity ratio was calculated using the following formula:

$$\begin{array}{c} \text{Bone marrow edema intensity ratio}=\\ \;\frac{\text{maximum signal intensity of bone marrow edema}}{\text{maximum signal intensity of muscle}} \end{array}$$

The degree of sclerosis (score range: 0–30) was determined on T1WI by evaluating 10 defined parcels, as shown in Figure 1b. Based on the degree of sclerosis, scoring per parcel was assigned as follows: 0 points: not cured; 1 point: sclerosis < 25%; 2 points: sclerosis < 50%; and 3 points: sclerosis > 50%.

Fatty degeneration (score range: 0–17) was evaluated on T1WI of the ACW, and 1 point each was given for the presence of fatty degeneration at 10 sites, as shown in Figure 1c. Depth (high‐signal area with a long diameter of ≥ 1 cm) was assessed at 7 sites: bilateral clavicle, first rib, manubrium of the sternum, and the midline junction between the manubrium and the body of the sternum (either the manubrium or the sternal body). A total score was calculated based on these assessments.

MRI examinations of the spine and sacroiliac joints were conducted to determine the spine SPARCC score and sacroiliac joint SPARCC score, respectively [23]. Evaluation of the spine SPARCC score (range 0–108) involved imaging 6 disc levels with signal abnormalities using STIR sequences across the entire spine. For each level, 3 consecutive sagittal cross‐sections showing the most prominent abnormalities were selected. Bone marrow signal was used as the reference signal. Scoring was based on the presence or absence of edema in 4 quadrants of the discovertebral unit (upper anterior endplate, lower anterior endplate, upper posterior endplate, and lower posterior endplate). If bone marrow edema was present, 1 point was given. Scoring was performed for each of the 3 consecutive sagittal slices, resulting in a maximum score of 12 per discovertebral unit. A high‐signal lesion as bright as cerebrospinal fluid was considered intense and given 1 point; an abnormal signal extending > 1 cm from the vertebral body surface was considered deep and also given 1 point. Hence, the total maximum score was 18 per unit, and with 6 discovertebral units evaluated, the total maximum score was 108. For evaluation of the sacroiliac joint SPARCC score (range: 0–72), STIR sequences were also used for imaging. The cross‐sections to be evaluated consisted of either 6 continuous oblique coronal sections showing the most signal abnormalities or 3 continuous anterior and 3 posterior oblique coronal sections from the sacral center on the sagittal section. Scoring was done as follows: the sacrum was divided into 4 sections (upper and lower, left and right), and the right and left ilium were divided into 2 sections (upper and lower). Each section was evaluated for the presence or absence of edema. If edema was present, 1 point was given; in addition, high‐signal lesions equal to vessels were considered intense and given 1 point. The presence of an abnormal signal extending ≥ 1 cm from the joint surface was considered deep and also given 1 point. This brought the maximum score for a single coronal slice to 24. After scoring each of the 3 slices, the cumulative maximum score was 72.

Plain radiography of the ACW was performed to calculate the osteosclerosis score (score range: 0–30). The sternum, sternoclavicular joints, and sternocostal joints (on both the left and right sides) were evaluated, with 1 point assigned for the presence of osteosclerosis at each site. Similarly, plain radiography of the spine and sacroiliac joints was performed to determine the modified Stoke Ankylosing Spondylitis Spinal Score (mSASSS) and modified New York (mNY) criteria score, respectively [24, 25].

The radiographic and MRI examinations used to assess inflammatory lesions and structural changes in the ACW, spine, and sacroiliac joints were interpreted via central reading. Two board‐certified musculoskeletal radiologists (with 15 and 10 years of experience, respectively) independently scored all imaging studies in a central reading room, blinded to the clinical data. In case of disagreement in interpretation, a third board‐certified musculoskeletal radiologist (with 20 years of experience) reviewed the images to make the final decision. For edema intensity values, if there was no significant discrepancy between the 2 evaluations, the average value was used. If a significant discrepancy existed, the measurement from the third radiologist was adopted.

### 2.3 Statistical Analysis

Correlations between clinical and MRI indicators, and between MRI and radiographic indicators, were evaluated. Continuous data were presented as means and standard deviations (SDs), and categorical data were presented as frequencies and percentages (%). Pearson's correlation coefficient (|*r*|) was used to assess the correlation between clinical and imaging indicators. *p*‐values were calculated based on the null hypothesis that no correlation existed. Since the objective of this study was to explore alternative markers of PAO disease activity, we adopted the criteria of a *p*‐value < 0.05 and |*r*| ≥ 0.7 as the standard for alternative markers based on previous literature [26]. Analyses were not adjusted for multiple comparisons because this was an exploratory study. Statistical analysis was performed using Base SAS Software System (SAS Institute, Cary, NC) version 9.4 and SAS/STAT (SAS Institute, Cary, NC) version 15.3.

## 3 Results

### 3.1 Patient Demographics and Baseline Characteristics

A total of 126 patients with PPP were included in the previous phase 3 trial [19]. Of these, 20 patients with concurrent PAO were included in this exploratory analysis. Table 1 presents the demographic and baseline characteristics of patients with PAO. The mean (SD) age of the patients was 56.6 (10.0) years. The majority of patients (*n* = 18; 90.0%) were female. At baseline, the mean (SD) PPPASI total score and ASDAS‐CRP score were 28.3 (13.7) and 2.6 (0.7), respectively. Among patients with PAO, none had a bone marrow edema extent or intensity score of 0. In contrast, 1 of 10 patients had a spine SPARCC score of 0, and 9 of 19 patients had a sacroiliac joint SPARCC score of 0.

**TABLE 1:** Demographic and baseline clinical characteristics of patients with PAO.

Characteristics	Patients (*N* = 20)
Age (years)	56.6 (10.0)
Sex, *n* (%)	
Female	18 (90.0)
Male	2 (10.0)
Weight (kg)	59.0 (13.0)
BMI (kg/m^2^)	23.3 (4.2)
Smoking habit, *n* (%)	
Smoker	11 (55.0)
Non‐smoker	9 (45.0)
PPPASI total score	28.3 (13.7)
ASDAS‐CRP score	2.6 (0.7)
CRP (mg/dL)	0.4 (0.6)
MRI indicators	
Bone marrow edema extent score on ACW MRI^a^	7.5 (4.1)
Bone marrow edema intensity ratio on ACW MRI^a^	3.4 (1.1)
SPARCC score on sacroiliac joint MRI^a^	4.6 (8.6)
SPARCC score on spine MRI^b^	24.2 (20.6)

*Note:* All values are presented as mean (SD), unless specified otherwise.

Abbreviations: ACW, anterior chest wall; ASDAS‐CRP, Ankylosing Spondylitis Disease Activity Score with CRP; BMI, body mass index; CRP, C‐reactive protein; MRI, magnetic resonance imaging; PAO, pustulotic arthro‐osteitis; PPPASI, Palmoplantar Pustulosis Area and Severity Index; SD, standard deviation; SPARCC, Spondyloarthritis Research Consortium of Canada.

^a^ *n* = 19, as 1 patient had missing data for MRI of the ACW and sacroiliac joints.

^b^ *n* = 10, as 10 patients had missing data for MRI of the spine.

### 3.2 Correlation Analysis Between Clinical and MRI Indicators

Table 2 shows the correlation analysis between the ASDAS‐CRP score and MRI indicators. A statistically significant positive correlation was observed between the ASDAS‐CRP score and the sacroiliac joint SPARCC score; however, the strength of the correlation did not meet the predefined criterion of |*r*| ≥ 0.7 for an alternative marker (|*r*| = 0.58; *p* = 0.0075). No clear associations were observed between individual components of the ASDAS‐CRP score and MRI indicators. Similarly, there was no clear association between the ASAS Health Index score and any MRI indicators. The physicians' NRS‐PAO activity score showed a statistically significant positive correlation with the sacroiliac joint SPARCC score, although the strength of the correlation did not meet the predefined criterion of |*r*| ≥ 0.7 for an alternative marker (|*r*| = 0.56; *p* = 0.0121). There was no clear association between the physicians' NRS‐PAO activity score and other MRI indicators. Similarly, the patients' NRS‐PAO pain score did not have a clear association with any MRI indicators.

**TABLE 2:** Correlation analysis of clinical indicators with MRI indicators.

Indicators	Bone marrow edema extent score on ACW MRI^a^		Bone marrow edema intensity ratio on ACW MRI^a^		SPARCC score on sacroiliac joint MRI^a^		SPARCC score on spine MRI^b^	
	|*r*|	*p*	|*r*|	*p*	|*r*|	*p*	|*r*|	*p*
ASDAS‐CRP score	−0.01	0.9807	0.15	0.5563	0.58	0.0075*	0.09	0.8021
Degree of back pain	0.14	0.5686	−0.28	0.2485	0.40	0.0951	−0.43	0.2206
Degree of general symptom intensity	0.14	0.5801	−0.06	0.7972	0.43	0.0632	−0.24	0.5126
Pain/swelling in joints other than the back	0.01	0.9593	0.21	0.3938	0.00	0.9857	−0.35	0.3315
Duration of morning stiffness	−0.09	0.7163	0.01	0.9800	0.13	0.5908	−0.29	0.4276
CRP (mg/dL)	0.06	0.8130	0.13	0.5934	0.35	0.1428	0.62	0.0559
ASAS Health Index score	−0.14	0.5703	−0.26	0.2954	0.30	0.2153	−0.60	0.0679
Physicians' NRS‐PAO activity score	0.07	0.7904	−0.16	0.5176	0.56	0.0121*	0.48	0.1661
Patients' NRS‐PAO pain score	0.03	0.9087	−0.27	0.2716	0.19	0.4365	−0.62	0.0577

*Note:* Pearson's correlation coefficient was calculated to assess the strength of correlation. *p*‐values were calculated based on the null hypothesis of no correlation. *r* = Pearson's correlation coefficient.

Abbreviations: ACW, anterior chest wall; ASAS, Assessment of SpondyloArthritis international Society; ASDAS‐CRP, Ankylosing Spondylitis Disease Activity Score with CRP; CRP, C‐reactive protein; MRI, magnetic resonance imaging; NRS‐PAO, Numeric Rating Scale of pustulotic arthro‐osteitis; SPARCC, Spondyloarthritis Research Consortium of Canada.

^a^ *n* = 19, as 1 patient had missing data for MRI of the ACW and sacroiliac joints.

^b^ *n* = 10, as 10 patients had missing data for MRI of the spine.

* A significant correlation was defined as |*r*| ≥ 0.7 and *p*‐value < 0.05.

### 3.3 Correlation Analysis Between Radiographic and MRI Indicators

The correlation analysis between radiographic and MRI indicators revealed a statistically significant positive correlation between the spine SPARCC score and the osteosclerosis score of the ACW (|*r*| = 0.70; *p* = 0.0218) and the mSASSS score of the spine (|*r*| = 0.93; *p* < 0.001) (Table 3 and Figure S1). Furthermore, there was a statistically significant positive correlation between the sacroiliac joint SPARCC score and the mNY reference score; however, the strength of the correlation did not meet the predefined criterion of |*r*| ≥ 0.7 for an alternative marker (|*r*| = 0.50; *p* = 0.0297).

**TABLE 3:** Correlation analysis of radiographic indicators with MRI indicators.

Imaging indicators	Bone marrow edema extent score on ACW MRI^a^			Bone marrow edema intensity ratio on ACW MRI^a^			SPARCC score on sacroiliac joint MRI^a^			SPARCC score on spine MRI^b^		
	*n*	|*r*|	*p*	*n*	|*r*|	*p*	*n*	|*r*|	*p*	*n*	|*r*|	*p*
*Radiographic indicators*												
ACW radiograph^c^												
Osteosclerosis score	19	0.12	0.6233	19	0.36	0.1290	19	0.29	0.2302	10	0.70*	0.0218*
Spine radiograph^b^												
mSASSS score	17	0.14	0.5971	17	−0.35	0.1736	17	−0.04	0.8860	8	0.93*	< 0.001*
Sacroiliac joint radiograph^c^												
mNY reference score	19	−0.10	0.6875	19	0.01	0.9604	19	0.50	0.0297*	10	0.07	0.8496
*MRI indicators*												
ACW MRI^a^												
Sclerosis score	19	0.08	0.7593	19	0.20	0.4193	19	0.20	0.4092	10	0.74*	0.0119*
Bone marrow edema extent score	—	—	—	19	0.04	0.8588	19	0.25	0.3031	10	0.29	0.4226
Bone marrow edema intensity ratio	19	0.04	0.8588	—	—	—	19	0.20	0.4169	10	−0.20	0.5878
Fatty degeneration score	19	−0.04	0.8736	19	−0.15	0.5378	19	−0.17	0.5009	10	Not calculated	Not calculated
Sacroiliac joint MRI^a^												
Sacroiliac joint SPARCC score	19	0.25	0.3031	19	0.20	0.4169	—	—	—	10	0.09	0.8129
Spine MRI^b^												
Spine SPARCC score	10	0.29	0.4226	10	−0.20	0.5878	10	0.09	0.8129	—	—	—

*Note:* Pearson's correlation coefficient was calculated to assess the strength of correlation. *p*‐values were calculated based on the null hypothesis of no correlation. *n* = number of patients assessed for each parameter; *r* = Pearson's correlation coefficient.

Abbreviations: ACW, anterior chest wall; mNY, modified New York criteria; MRI, magnetic resonance imaging; mSASSS, modified Stoke Ankylosing Spondylitis Spinal Score; SPARCC, Spondyloarthritis Research Consortium of Canada.

^a^ 1 patient had missing data for MRI of the ACW and sacroiliac joints.

^b^ 10 patients had missing data for MRI of the spine, except for the mSASSS score, for which 2 additional patients had missing spine radiograph data.

^c^ Plain radiographs of the ACW and sacroiliac joints were available for 20 patients.

* A significant correlation was defined as |*r*| ≥ 0.7 and *p*‐value < 0.05.

The correlation analysis between different MRI indicators showed a positive correlation between the spine SPARCC score and the sclerosis score on the ACW (|*r*| = 0.74; *p* = 0.0119) (Table 3 and Figure S1). In contrast, no clear association was observed between other MRI indicators, including bone marrow edema extent (by ACW MRI) and the sclerosis score, fatty degeneration score, sacroiliac joint SPARCC score, and spine SPARCC score. Notably, there was no clear association between the bone marrow edema extent score and the bone marrow edema intensity ratio (|*r*| = 0.04; *p =* 0.8588) (Table 3 and Figure S2).

## 4 Discussion

We performed an exploratory analysis using data from a subgroup of patients with PPP diagnosed with concurrent PAO enrolled in a phase 3 trial of brodalumab [19] to explore candidate indicators of PAO disease activity. The results of the correlation analysis between MRI and clinical indicators demonstrated that none of the assessed clinical indicators showed a clear association with MRI indicators, suggesting that currently available clinical indicators may not adequately reflect disease activity in PAO.

One of the major challenges in PAO diagnosis is the lack of established evaluation methods. As a result, various subjective and objective tools have been used to evaluate PAO disease activity in previous studies. Currently, MRI is an important diagnostic tool used in spondyloarthritis due to its ability to detect both chronic and active inflammatory changes in the ACW and spine [27, 28, 29]. To the best of our knowledge, this is the first exploratory analysis to develop an MRI‐based scoring system specifically targeting ACW involvement in patients with PPP‐associated PAO using clinical trial data. Given that ACW involvement is the predominant and often the most symptomatic site in PAO, a structured method of evaluation focused on this region could be particularly relevant in dermatology practice.

Recently, Yu et al. characterized ACW lesions using MRI in 71 patients with SAPHO syndrome [30]. It was observed that all patients exhibited abnormalities in the ACW, with the majority of lesions located in the sternocostoclavicular region (93.0%). Furthermore, MRI detected acute inflammatory changes such as bone marrow edema and synovitis in 88.7% and 90.1% of patients, respectively. It was confirmed that all patients had experienced anterior chest pain during the course of their disease, although it was not confirmed whether anterior chest pain was present at the time of MRI imaging. In the present analysis, all patients with PAO had bone marrow edema in the ACW, whereas some scored 0 on the spine or sacroiliac joint SPARCC scores, suggesting an absence of edema at those sites. A previous study also showed that asymptomatic lesions that are not clinically apparent can be detected in the SAPHO/chronic nonbacterial osteitis region using whole‐body MRI or bone scintigraphy, suggesting that imaging findings and symptoms may not always correspond one‐to‐one [31]. Therefore, it seems more appropriate to interpret MRI‐detected ACW bone marrow edema not as a direct surrogate marker of pain, but rather as a candidate imaging biomarker reflecting local inflammatory activity and based on this we developed an evaluation method for quantifying disease activity using MRI. Interestingly, no clear association was observed between the bone marrow edema extent score and the bone marrow edema intensity ratio in the ACW on MRI. This may reflect the underlying differences in the temporal dynamics of inflammatory resolution. Typically, bone marrow edema signals may persist even after the signal intensity has diminished. In fact, it is difficult to distinguish between simple cell swelling due to extracellular fluid accumulation and inflammation from fresh granulation tissue based solely on MRI findings [32]. Although this analysis did not determine which indicator better reflects PAO activity or is more suitable for treatment monitoring, it may be advisable to monitor both scores in clinical settings.

In this analysis, we investigated whether clinical indicators reflect PAO disease activity by analyzing their correlation with bone marrow edema intensity and extent scores. No clear association was observed between the quantitative scores for the intensity and extent of bone marrow edema in the ACW, as measured by MRI, and any of the clinical indicators. This may be because the available clinical indicators do not specifically assess the ACW, despite it being the primary site affected in PAO. In the future, MRI could be combined with PAO‐specific clinical indicators capable of predicting active inflammatory lesions in the ACW to aid in PAO diagnosis.

MRI provides detailed imaging, whereas radiography is more accessible and cost‐effective. Although the difficulty of detecting early PAO lesions on radiography is well known [3], its utility in assessing disease activity has not been previously reported. In the present analysis, no clear association was found between ACW bone marrow edema and radiographic findings, suggesting that plain radiography, which can only detect structural changes, is insufficient to estimate disease activity in PAO. On the other hand, a higher spine SPARCC score was associated with radiographic abnormalities of the ACW and spine. Therefore, MRI of the ACW and spine should be considered when radiographic abnormalities are present, or when clinical suspicion exists despite unremarkable radiographs, to enable early detection of PAO.

Some limitations of this analysis should be acknowledged. First, this was a post hoc exploratory analysis of patients with comorbid PAO from a clinical trial originally designed for patients with PPP [19], resulting in a small sample size (*n* = 20). In addition, spinal MRI was performed on an optional basis, leading to a further reduction in the number of available imaging datasets and the potentially introducing selection bias. As a result, the statistical power was limited; therefore, the absence of robust correlations should not be interpreted as evidence of no true association. Future studies with larger sample sizes are needed to further assess and validate disease activity indicators in patients with PAO. Second, the lack of established quantification methods for evaluating ACW involvement using MRI represents another limitation of the present analysis. Third, the correlation analyses were not adjusted for potential confounding factors or multiple testing. Given the small dataset, this may have increased the risk of both false‐positive and false‐negative findings. Finally, the post hoc design and inclusion of patients from a clinical trial population may limit the generalizability of these findings.

## 5 Conclusions

Since PPP is primarily managed in dermatology settings, dermatologists are often the first to recognize musculoskeletal symptoms suggestive of PAO. A standardized imaging‐based assessment of ACW involvement may therefore facilitate earlier referral and multidisciplinary management. In this exploratory study, a quantitative evaluation method for assessing active and structural lesions using MRI was developed to evaluate PAO disease activity in the ACW. Although no clear association was identified between clinical parameters and the developed MRI indicators, the results of this analysis suggest that MRI may play a central role in the assessment of PAO. Additionally, this study highlights the need to establish new clinical indicators specifically suited for evaluating PAO disease activity.

## Funding

This study was sponsored by Kyowa Kirin Co., Ltd.

## Ethics Statement

The study was approved by the institutional review board at Keio University School of Medicine (approval number: 20241098) and the Research Ethical Review Committee of Kyowa Kirin Co., Ltd. It was conducted in accordance with the ethical principles of the Declaration of Helsinki, the Council for International Organizations of Medical Sciences (CIOMS) International Ethical Guidelines, the International Council for Harmonization of Technical Requirements for Pharmaceuticals for Human Use Good Clinical Practice (ICH‐GCP) guidelines, and other applicable laws and regulations. Written informed consent was obtained from all patients for participation in the phase 3 clinical trial (NCT04061252). Although this secondary analysis falls within the scope of the original consent, additional information regarding data use was made publicly available via an opt‐out notice, thereby allowing participants the opportunity to decline inclusion in this analysis.

## Conflicts of Interest

T.N., M. Arai, and J.O. have received honoraria from Kyowa Kirin Co., Ltd. M.U.‐Y. and Y. Kanai are employees of Kyowa Kirin Co., Ltd. M.H., M. Akiyama, Y. Kaneko, and M.J. have no conflicts of interest to declare.

## Supporting information


**Figure S1:** Correlation analysis between (a) SPARCC score on spine MRI and osteosclerosis score on ACW radiograph, (b) SPARCC score on spine MRI and mSASSS score on spine radiograph, and (c) SPARCC score on spine MRI and sclerosis score on ACW MRI.
**Figure S2:** Correlation analysis between bone marrow edema extent score and bone marrow edema intensity ratio on ACW MRI.

## Data Availability

The datasets generated and/or analyzed during the current study will be available in the Vivli repository (https://vivli.org/ourmember/kyowa‐kirin/), provided that the conditions for data disclosure specified in the policy section of the Vivli website are satisfied.
