# Supplementary material for: Exploratory MRI and Clinical Marker Analysis of Pustulotic Arthro‐Osteitis Disease Activity From a Phase 3 Brodalumab Trial
Source: J Dermatol. 2026 Aug 2;53(10):1448–56. doi: 10.1111/1346-8138.70425 (PMC13646863; doi:10.1111/1346-8138.70425)
Supplement: Supplementary file 1 [file JDE-53-1448-s001.docx]

**SUPPLEMENTARY MATERIAL**

**Figure S1.** Correlation analysis between a) SPARCC score on spine MRI and osteosclerosis score on ACW radiograph, b) SPARCC score on spine MRI and mSASSS score on spine radiograph, and c) SPARCC score on spine MRI and sclerosis score on ACW MRI

ACW, anterior chest wall; MRI, magnetic resonance imaging; mSASSS, modified Stoke Ankylosing Spondylitis Spinal Score; SPARCC, Spondyloarthritis Research Consortium of Canada.

n=number of patients assessed for each parameter; r: Pearson’s correlation coefficient.

Pearson’s correlation coefficient was calculated to assess the strength of correlation. *P*-values were calculated based on the null hypothesis of no correlation. A significant correlation was defined as |r| ≥0.7 and *P*-value <0.05.

n=10 as 10 patients had missing data for MRI of the spine, except for the mSASSS score (n=8), for which 2 additional patients had missing spine radiograph data.

**Figure S2.** Correlation analysis between bone marrow edema extent score and bone marrow edema intensity ratio on ACW MRI

ACW, anterior chest wall; MRI, magnetic resonance imaging.

n=number of patients assessed for each parameter; r: Pearson’s correlation coefficient.

Pearson’s correlation coefficient was calculated to assess the strength of correlation. *P*-values were calculated based on the null hypothesis of no correlation. A significant correlation was defined as |r| ≥0.7 and *P*-value <0.05.

n=19 as 1 patient had missing data for MRI of the ACW.
